# Case Report: Early ^68^Ga-PSMA-PET Metabolic Assessment and Response to Systemic Treatment for First-Line Metastatic Clear Cell Renal Cell Carcinoma; About Two Clinical Cases

**DOI:** 10.3389/fonc.2021.782166

**Published:** 2021-12-07

**Authors:** Emmanuel Seront, Renaud Lhommel, Bertrand Tombal

**Affiliations:** ^1^Division of Medical Oncology, Institut de Recherche Clinique (IREC), Cliniques Universitaires Saint Luc, Brussels, Belgium; ^2^Division of Nuclear Medicine, Institut de Recherche Clinique (IREC), Cliniques Universitaires Saint Luc, Brussels, Belgium; ^3^Division of Urology, Institut de Recherche Clinique (IREC), Cliniques Universitaires Saint Luc, Brussels, Belgium

**Keywords:** renal cell carcinoma, axitinib, pembrolizumab, gallium, metastatic disease, PSMA

## Abstract

Early evaluation of response to anticancer treatment in metastatic renal cell carcinoma (RCC) is challenging as responses are sometimes delayed, as mixed responses can occur, and as conventional imaging have some limitations. As PSMA has been previously identified in neovasculature of clear cell RCC (ccRCC), ^68^Ga-PSMA-Positron Emitted Tomography (PET) could appear as an interesting tool to evaluate therapeutic response. We describe the association of an early decrease in ^68^Ga metabolism (at 8 weeks after treatment onset) and further radiological response (at 12 weeks after treatment onset) to treatment in two patients with different sensitivity to axitinib–pembrolizumab combination. Interestingly, one of these patients presented an initial progressive disease on pembrolizumab alone and a subsequent response to axitinib alone in the disease course; these response profiles were associated with absence of decrease and subsequent decrease in the ^68^Ga metabolism, respectively. Even if further prospective trials are needed, ^68^Ga-PSMA-PET may appear as a promising way for early prediction of response to ccRCC systemic treatment.

## Introduction

Renal cell carcinoma (RCC) is the 7th most common malignancy with 338,000 new cases and 144,000 related deaths worldwide yearly; clear cell histology (ccRCC) accounts for 80% of the cases. One-third of RCC patients present at diagnosis with metastatic disease, and among patients with initially localized disease, a significant proportion will develop metastases (1–3). Antiangiogenic Tyrosine Kinase Inhibitors (TKI) and Immune Checkpoint Inhibitors (ICI) are widely used in therapeutic strategy for metastatic RCC; however, efficacy remains modest, with a response rate not exceeding 30% and a 5-year survival rate not exceeding 20% (4–6). More recently, the association of ICI (pembrolizumab or avelumab) and TKI (axitinib) or the combination of two ICIs (nivolumab + ipilimumab) have been established as the new first-line standard treatment, increasing the response rate to 50–60% and improving the progression free survival and the overall survival compared to sunitinib (7–9).

Imaging evaluation is challenging in RCC; standard modalities such as computed tomography (CT) and bone scan are sometimes unable to detect metastatic dissemination or characterize suspect distant lesions. Furthermore, radiological response on anticancer treatment can be difficult to evaluate; response pattern observed with TKI or ICIs can include a prolonged disease stabilization before an ultimate tumor shrinkage, an initial increase in the tumor burden or a mixed response with new lesions. In addition, an early identification of therapeutic inefficacy could prevent the continuation of this treatment (10).

^68^Gallium (^68^Ga)-Prostate-Specific Membrane Antigen (PSMA)-Positron Emitted Tomography (PET) has been shown to be more sensitive and specific than CT and bone scan in prostate cancer. Since PSMA expression has been previously identified in the neovasculature of benign and malignant tumors including RCC, we have investigated ^68^Ga-PSMA-PET in two ccRCC metastatic patients treated with TKI + ICI combination (11–17). To our knowledge, whether an early change in ^68^Ga metabolism could predict response to these treatments remains unknown.

## Case Description

**Figure 1:**
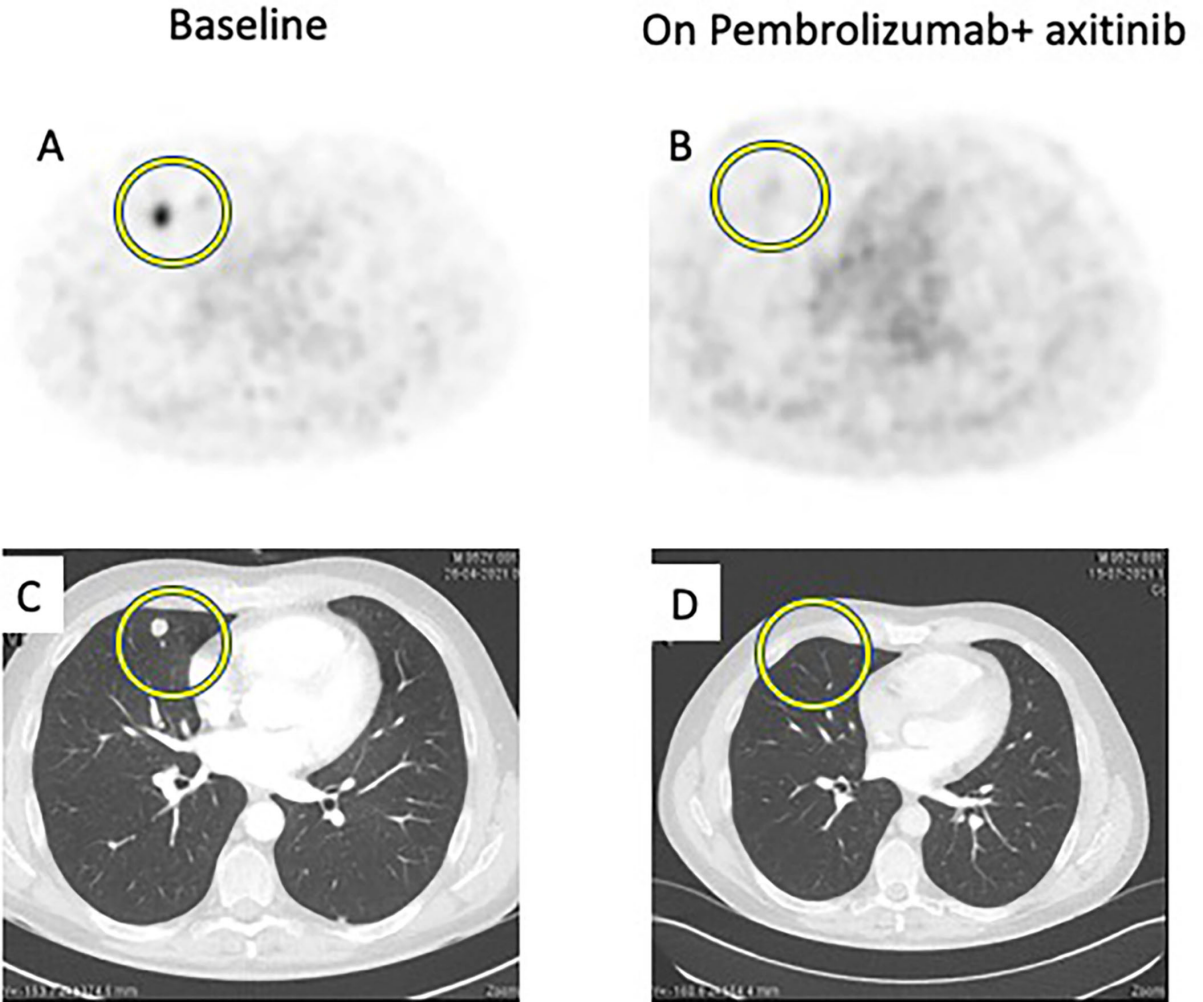
Comparison between baseline imaging and on-treatment imaging. ^68^GaPSMA-PET at baseline **(A)** and at 8-week **(B)** showing decrease of metabolism (yellow circle). Thoracic CT Scan at baseline **(C)** and at 12-week **(D)** showing disappearance of pulmonary lesions (yellow circle).

First case: an early decrease of ^68^Gallium metabolism and further partial response on CT scan (**Figure 1**).

A 52-year-old male patient was diagnosed in January 2021 with a 10-centimeter left renal tumor. Baseline imaging work-up showed 3 millimetric bilateral lung lesions. Nephrectomy was performed, confirming a 11-centimeter ccRCC with tumor necrosis, grade ISUP 3 (International Society of Urological Pathology), and renal vein thrombus; the final staging was pT3a based on TNM classification (8th edition). No adjuvant treatment was started. In May 2021, a thoraco-abdominal CT showed an increase in size and number of lesions. ^68^Ga-PSMA-PET showed 10 metabolic bilateral pulmonary lesions, ranging from 6 to 14 mm in size, and from 2.3 to 9.2 in SUVmax; there was no other detected lesion. Blood test was normal, namely, hemoglobin, calcium, and lactate deshydrogenase (LDH). This patient was classified as an intermediate risk group following the International Metastatic RCC Database Consortium (IMDC) risk group classification as the interval between diagnosis and treatment onset was inferior to 12 months. We started at this time association of pembrolizumab (200 mg every three weeks) + axitinib (5 mg bid). Eight weeks after treatment onset, a new ^68^Ga-PSMA-PET CT showed disappearance of ^68^Ga metabolism in pulmonary lesions and a decrease in the size of all lesions (partial response following RECIST criteria). This response was maintained on the CT scan performed 12 weeks after treatment onset (**Figure 1**).

**Figure 2:**
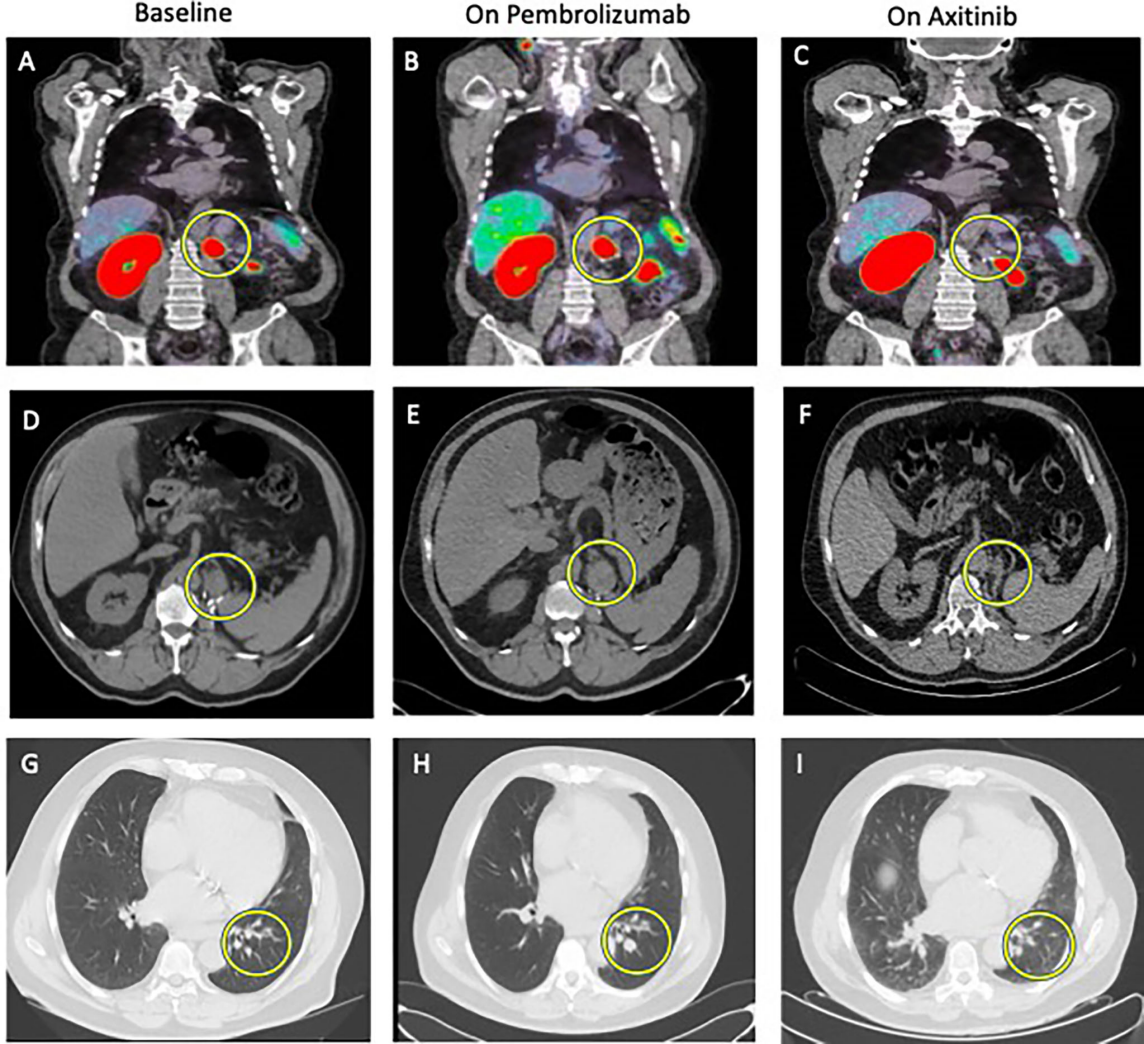
Comparison between baseline imaging and on-treatment imaging. ^68^GaPSMA-PET at baseline **(A)** and at 8-week on pembrolizumab alone **(B)** showing absence of metabolism decrease (yellow circle) and at 16-week on axitinib alone **(C)** showing disappearance of metabolism (yellow circle). Other metabolic lesion was not cancer lesion, but normal kidney (left) and physiological bowel metabolism (right on the picture). Abdominal CT Scan at baseline **(D)**, at 12-week **(E)** showing increase in size of the lesion at the nephrectomy site (yellow circle) and at 16-week **(F)** showing partial response. Thoracic CT Scan at baseline **(G)**, at 12-week **(H)** showing increase in size of pulmonary lesions (yellow circle) and at 16-week **(I)** showing partial response.

Second case: An absence of decrease of ^68^Gallium metabolism and further radiological progression on CT scan (**Figure 2**).

A 72-year-old male patient was treated in 2018 with nephrectomy for a 6-centimeter ISUP 2 ccRCC with renal vein thrombus, classified as pT3a N0M0. In February 2021, a resurgence of the disease was diagnosed with a 26-millimeter tumor lesion in the nephrectomy site and three infra-centimetric lesions in the left inferior pulmonary lobe. ^68^Ga-PSMA-PET showed intense metabolism of the local lesion (SUVmax 21.8) and moderate metabolism of the three pulmonary lesions (SUVmax ranging from 2.6 to 4.7). Blood tests (renal function, calcium, LDH, and liver tests) were normal except a grade 1 anemia. This patient was thus classified as an IMDC intermediate risk group. Systemic treatment with pembrolizumab (200 mg every three weeks) plus axitinib (5 mg bid) was started but axitinib had to be interrupted after 3 weeks due to the Common Terminology Criteria for Adverse Events (CTCAE v6) grade 3 nephrotic syndrome. Eight weeks after treatment onset, ^68^Ga-PSMA-PET-CT showed an absence of decrease in ^68^Ga metabolism in both local lesion and pulmonary lesions and the absence of decrease in lesions size (stable disease based on the RECIST criteria). The 12-week CT scan confirmed a disease progression following the RECIST criteria with an increase in the size of the pulmonary lesions (from 5 to 8 mm, from 4.5 to 9 mm and from 8 to 15 mm) and of the lesion in the nephrectomy site (from 2 to 38 mm). At this time, pembrolizumab alone was stopped and axitinib was reintroduced at 5 mg daily. Four weeks later (16 weeks after treatment initiation), ^68^Ga-PSMA-PET-CT showed a disappearance of ^68^Ga metabolism in all metastatic lesions; that was associated with a significant decrease in size of the local lesions (from 38 to 25 mm) and of the pulmonary lesions (from 8 to 4 mm, from 9 to 2 mm and from 15 to 6 mm) (**Figure 2**).

**Figure 3:**
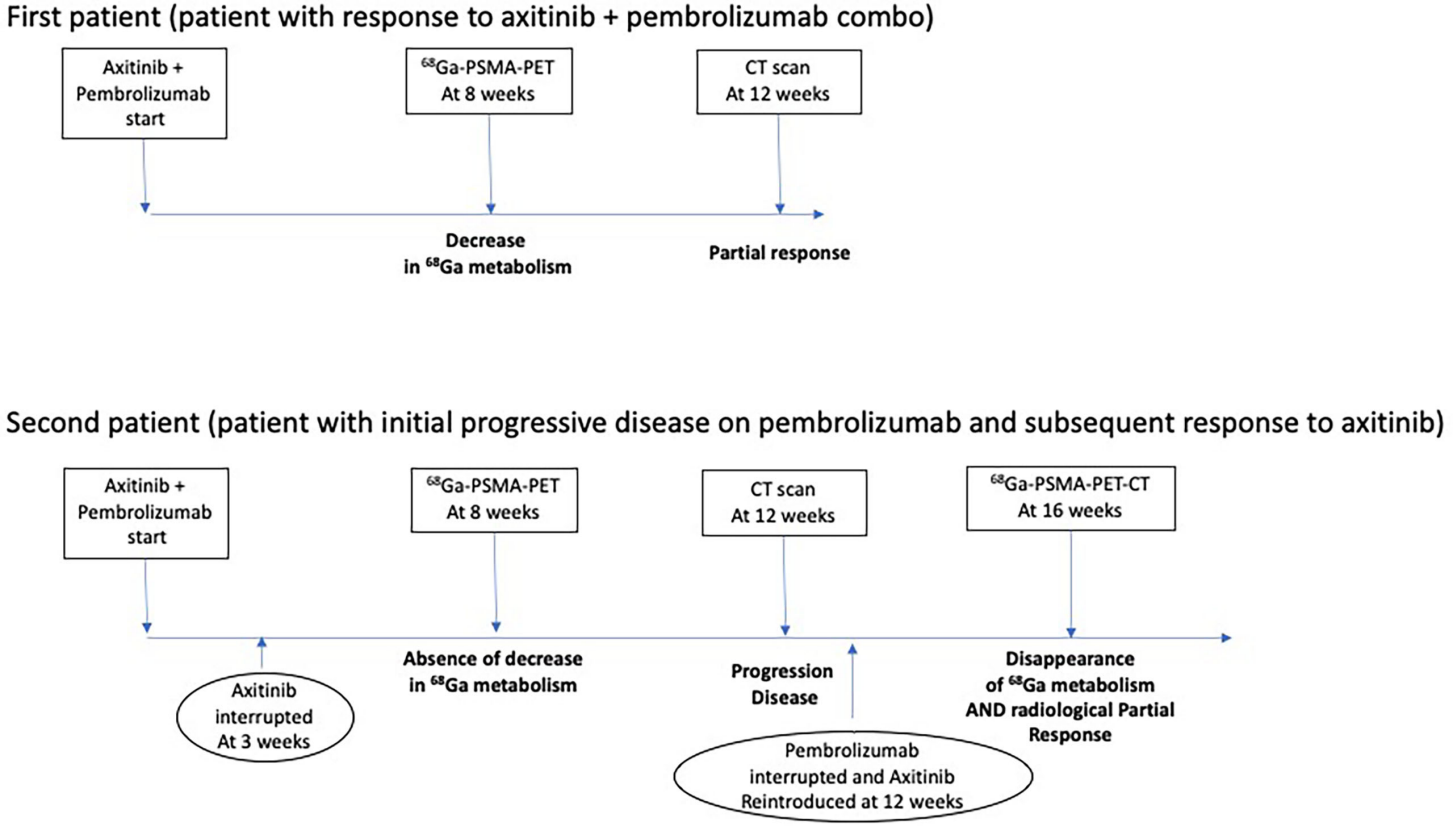
Timeline with imaging and response to therapies in the two clinical cases.

Figure showcasing a timeline with imaging and response to treatment in the two clinical cases (**Figure 3**).

## Discussion

The type II transmembrane glycoprotein PSMA is significantly overexpressed in most prostate cancer cells and is associated with increasing tumor grade and stage. ^68^Ga-PSMA PET-CT imaging demonstrated superior sensitivity and specificity compared to conventional imaging (thoraco-abdominal CT and bone scan) in primary, metastatic, and biochemically recurrent prostate cancer (18–20).

Interestingly, the increasing use of PSMA PET-CT imaging in prostate cancer has revealed PSMA ligand uptake in multiple non-prostatic benign and malignant diseases (21). Renal cell carcinoma (RCC) is a heavily vascularized tumor with well-documented PSMA expression in the neovasculature (22)

^68^Ga-PSMA-PET-CT is an emerging imaging modality in the ccRCC management. Different retrospective studies showed that ^68^Ga-PSMA-PET-CT was able to identify aggressive pathological features (high grade or sarcomatoid features) of ccRCC in the preoperative setting (23, 24) and to identify metastatic lesions or synchronous primary that were not detected on standard imaging (25). Furthermore, Rhee et al. prospectively showed in 10 RCC patients that the ^68^Ga-PSMA-PET-CT had a stronger detection rate of metastases compared to CT scan, with less false negative lesions; this resulted in a change in the therapeutic management in the two patients (26). However, the role of ^68^Ga-PSMA-PET-CT in early evaluation of response to treatment (TKI and ICI) remains unknown.

We report two types of radiological responses in the two ccRCC patients treated with TKI-ICI association on the 12-week standard imaging: a partial response and a progression disease based on the RECIST criteria. Interestingly, we showed that the 8-week ^68^Ga metabolism change may be correlated with the 12-week radiological response. In the first patient, the decrease of ^68^Ga metabolism was associated with the decrease in size and number of lesions. In the second patient, the absence of decrease in ^68^Ga metabolism on pembrolizumab was associated with the further progression in size of described lesions. Importantly, in this last patient, the introduction of a more effective treatment (axitinib), resulted in the early disappearance of ^68^Ga metabolism, confirming the association between ^68^Ga metabolism and response to treatment.

These associations between metabolic and radiological responses appear as promising in the early evaluation of response to ICI-combined treatments; radiological responses are sometimes delayed, appearing in some cases after many months, which renders the therapeutic evaluation challenging. Interpretation of mixed response, pseudo-progression and bone lesions evolution appears also difficult for clinicians and radiologists in the assessment of response.

Early assessment of ^68^Ga metabolism of ccRCC lesions could be helpful in management of these patients; early detection of non-responding patients could prevent continuation of inefficient and potentially toxic treatment. In the era of combinations (TKI + ICI or ICI + ICI), the absence of predictive biomarker leads clinicians to start association of treatments; early prediction of response could help to adapt our management by starting monotherapy and assessing rapidly the metabolic response. Furthermore, this could also open new strategies including Lutetium-based treatment, particularly in patients with low tumor burden in which close surveillance attitude is adopted.

This report is only a description of two ccRCC patients that were treated with a similar combination. Even if TKI had to be stopped early in one of these patients, its further reintroduction allowed this patient to be the control of himself in terms of treatment efficacy assessment. Further prospective trials should be done with higher number of patients, similar characteristics (treatment schemes, IMDC risk group).

If 68Ga-PSMA-PET appears as a promising method for staging and characterizing RCC, it may also have a role in the early assessment of treatment efficacy in metastatic setting.

## Patient Perspective

The first patient: “having a good tool for rapidly predicting response to the treatment prevents the stress for waiting during three months whether there is a response or not to treatment”. Of course, starting a treatment with potential toxicities in patients remains a challenge for clinicians. Having early tools to confirm efficacy will help the clinician to continue the same treatment or change towards another subsequent treatment.

The second patient: “choosing a treatment is challenging for the practician as we don’t know whether this treatment will be efficient. In my case, after failure of pembrolizumab, it was important for me to rapidly known whether axitinib was efficient”. In this case, pembrolizumab was not continued as PSMA-PET did not show any activity of this agent, which was confirmed later by CT scan.

## Data Availability Statement

The original contributions presented in the study are included in the article/supplementary material. Further inquiries can be directed to the corresponding author.

## Ethics Statement

Written informed consent was obtained from the patient for the publication of any potentially identifiable images or data included in this article.

## Author Contributions

ES followed the patient, administered treatment, and wrote the manuscript. RL followed the patient, evaluated response with PSMA-PET and wrote the manuscript. BT followed the patient, administered treatment, and wrote the manuscript. All authors contributed to the article and approved the submitted version.

## Conflict of Interest

The authors declare that the research was conducted in the absence of any commercial or financial relationships that could be construed as a potential conflict of interest.

## Publisher’s Note

All claims expressed in this article are solely those of the authors and do not necessarily represent those of their affiliated organizations, or those of the publisher, the editors and the reviewers. Any product that may be evaluated in this article, or claim that may be made by its manufacturer, is not guaranteed or endorsed by the publisher.
